# Role of testosterone to estradiol ratio in predicting the efficacy of recombinant human chorionic gonadotropin and testosterone treatment in male hypogonadism

**DOI:** 10.20945/2359-3997000000399

**Published:** 2021-09-29

**Authors:** Mehmet Çelik, Serhat Özçelik, Süleyman Baş, Mehmet Sariaydin, Melike Özçelik, Hulya Gozu

**Affiliations:** 1 Trakya University Faculty of Medicine Department of Endocrinology and Metabolism Edirne Turkey Department of Endocrinology and Metabolism, Trakya University Faculty of Medicine, Edirne, Turkey; 2 Adiyaman Training and Research Hospital Department of Endocrinology and Metabolism Adiyaman Turkey Department of Endocrinology and Metabolism, Adiyaman Training and Research Hospital, Adiyaman, Turkey; 3 Haydarpaşa Numune Training and Research Hospital Department of Internal Medicine Istanbul Turkey Department of Internal Medicine, Haydarpaşa Numune Training and Research Hospital, Istanbul, Turkey; 4 University of Health Sciences Umraniye Training and Research Hospital Department of Internal Diseases Turkey University of Health Sciences, Umraniye Training and Research Hospital, Department of Internal Diseases, Turkey; 5 Marmara University Faculty of Medicine Department of Endocrinology and Metabolism Istanbul Turkey Department of Endocrinology and Metabolism, Marmara University Faculty of Medicine, Istanbul, Turkey

**Keywords:** Hypogonadism, testosterone to estradiol ratio, morning erection

## Abstract

**Objective:**

We aimed to investigate the role of testosterone to estradiol ratio in predicting the effectiveness of human chorionic gonadotropin and testosterone treatments in male hypogonadism.

**Materials and methods:**

Thirty-six male patients with hypogonadotropic hypogonadism were included in the study. Seventeen (47.2%) patients received weekly recombinant human choriogonadotropin alpha (hCG) treatment (group-1) and 19 (52.8%) received testosterone replacement therapy (T treatment) every 21 days (group-2). Under these treatments, adequate frequency of morning erection (≥3/week), testosterone to estradiol ratio (T/E), and testicular volume changes were analyzed.

**Results:**

The mean age of the patients was 28.5 ± 8.7 years. When the frequency of morning erection (≥3/week) was specified as adequate, the cut-off value for effective T/E ratio was found to be 12.0 (sensitivity 93.8%, specificity 90.0%). There was no significant difference between the treatment groups in terms of total testosterone levels, T/E ratio, or frequency of morning erections (≥3/week) (p > 0.05). However, there was a statistically significant difference between the groups in terms of median left-right testicular volume in favor of group-1 (p < 0,05).

**Conclusion:**

In patients with hypogonadism who are under treatment, elevated estradiol-induced erectile dysfunction symptoms may persist even if serum testosterone levels are normal. Testosterone to estradiol ratio can be used as a predictive value in the effective treatment of hypogonadotropic hypogonadism with hCG and T.

## INTRODUCTION

Hypogonadism is a gonadal dysfunction that can occur as a result of disruption in one or both of the gametogenesis and gonadal hormone secretions ( 1 ). Gonadal functions are under the control of hypothalamic gonadotropin-releasing hormone (GnRH), follicle-stimulating hormone (FSH), and luteinizing hormones (LH). LH stimulation induces testosterone production in the testicle and FSH stimulates spermatogenesis ( 2 ). Dysfunction due to gonadal failure is called primary hypogonadism (hypergonadotropic hypogonadism) and hypogonadism caused by the lack of hypophyseal hormones is known as secondary hypogonadism (hypogonadotropic hypogonadism).

The diagnosis of androgen insufficiency in men is considered in the presence of low testosterone levels consistent with symptoms and signs of hypogonadism. The most common symptoms and signs are: decreased libido, sexual activity, and spontaneous erection; gynecomastia, decreased body hair or shaving frequency, small or shrunken testicle, infertility, and absence or low count of sperms ( 1 , 3 ). Today, expanded knowledge about normal male sexual function and the causes of sexual impairment has led to the development of effective treatments. The main goals of treatment in male hypogonadism are to correct the symptoms of testosterone deficiency and maintain secondary sex characteristics. In healthy adult men, the target of the treatment is to keep the testosterone level at the lower limit of the normal range (280-300 ng/dL [9.7-10.4 nmol/L]) ( 4 ). Hormone replacement is the preferred treatment option in hypogonadotropic hypogonadism ( 5 ) and has evolved to comprise various options, including recombinant human FSH, recombinant human LH, and pulsatile gonadotropin-releasing hormone (GnRH), in addition to human chorionic gonadotropin (hCG), human menopause gonadotropin (hMG), and testosterone (T) treatments ( 6 , 7 ). According to the current state of the literature, the effectiveness of these different treatment strategies remains controversial ( 8 , 9 ). Besides, it is not clear what role estrogen hormone plays as a factor in regulating sexual dysfunction in men. In this regard, the effect of imbalance between testosterone and estradiol on sexuality has not yet been clearly determined ( 10 ). It has been suggested that rather than the direct effect of estrogens on erectile dysfunction and decreased libido, the testosterone-estradiol (T/E) ratio may be correlated with these symptoms ( 11 ). The aim of this study is to evaluate the role of T/E ratio in estimating the effectiveness of hCG and T treatment in male hypogonadism.

## MATERIALS AND METHODS

### Study population

**Figure 1:**
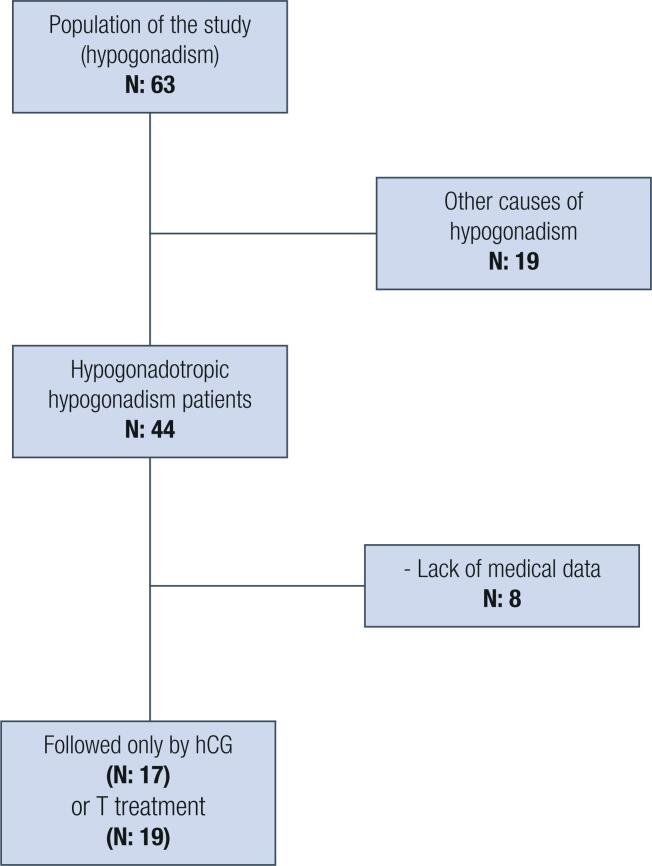
Flow chart of the study.

This retrospective observational study was carried out between October 2016 and July 2018 at the Department of Endocrinology and Metabolism Diseases at Adiyaman University Training and Research Hospital, after approval by the local Institutional Review Boards of participating centers. We analyzed 63 patients with hypogonadism, 27 of whom were excluded ( Figure 1 ). Finally, the data of 36 male patients diagnosed with hypogonadotropic hypogonadism were evaluated.

hCG: human chorionic gonadotropin; T level: total testosterone level.

### Etiologies of hypogonadotropic hypogonadism

The diagnosis of hypogonadotropic hypogonadism was determined according to clinical history, low gonadotropin and total testosterone levels for age, and low responses to GnRH stimulation test (LH-RH Ferring^®^ 0.1 mg/mL).

Kallmann syndrome was diagnosed based on reduced or lack of sense of smell. Two of our patients were accepted as Kallmann syndrome: 1 as hypogonadism secondary to craniopharyngioma, 4 as hypogonadism secondary to pituitary adenoma operation, and the remaining 29 as congenital hypogonadotropic hypogonadism. Besides, two of our patients were siblings.

Patients with BMI over 25 kg/m^2^ have no response to the GnRH stimulation test. Therefore, functional hypogonadism was not considered in this group of patients.

The inclusion criteria were (a) being aged 18 years and over (b) being clinically, biochemically, and radiologically diagnosed with hypogonadotropic hypogonadism (diagnosis of hypogonadotropic hypogonadism was made with detection of serum total testosterone levels (range: 218-915 ng/dL) within the lower limits of normal or even lower, and FSH (range:1.5-8.6 mIU/mL) and LH (range:1.7-12.4 mIU/mL) levels as low or discordantly normal), (c) and receiving treatment only with hCG or T.

The exclusion criteria were (a) medical data deficiency (b) medication that affects testosterone levels (c) hypergonadotropic hypogonadism (Klinefelter syndrome, bilateral anorchia or cryptorchidism, chemotherapeutic agents, varicocele, and idiopathic), (d) erectile dysfunction (vasculogenic, neurogenic, local penile factors, drug-related, psychogenic), (e) or other hormone disorders (hyperprolactinemia, hyper-hypothyroidism and hyper-hypocortisolism).

### Treatment and follow-up

After the diagnosis of hypogonadotropic hypogonadism, the patients were informed about the options of T, hCG, or pulsatile GnRH treatments. Recombinant hCG treatment was started for 17 patients who wanted to have children at the dose of 1000-2000 IU, 2-3 times a week. Since 19 patients did not want fertility, intramuscular 250 mg testosterone (testosterone isocaproate 60 mg + testosterone phenylpropionate 60 mg + testosterone propionate 30 mg + testosterone decanoate 100 mg) was commenced to apply every 21 days. During follow-up, the medication dose was titrated until a sufficient level of testosterone was reached ( Table 1 ).

**Table 1:** Demographic, anthropometric, and biochemical characteristics of patients

Variable		Value (n: 36)
Age (year) *		28.5 ± 8.7
BMI (kg/m^2^) *		25.5 ± 2.7
Hematocrit (%) *		44.0 ± 3.4
FSH (mIU/mL) †		1.6 [2.9]
LH (mIU/mL) †		0.9 [1.7]
ACTH (pg/mL) *		23.9 ± 7.2
Cortisol (μg/dL) †		11.6 [6.2]
TSH (μIU/mL) †		1.8 [1.3]
Prolactin (ng/mL) *		9.8 ± 5.5
Mean testicular volume (mL) (hCG treatment) (n: 17) †	Left	1.6 [1.9]
	Right	1.6 [2.5]
Mean testicular volume (mL) (T treatment) (n: 19) †	Left	1.1 [6.3]
	Right	1.0 [5.8]
Total testosterone (ng/mL) †		50.5 [51]
Estradiol (pg/mL) *		32.0 ±12

BMI: body mass index; FSH: follicle-stimulating hormone; LH: luteinizing hormone; ACTH: adrenocorticotropic hormone; TSH: thyroid-stimulating hormone; hCG: human chorionic gonadotropin; T level: total testosterone level.

* Data are presented as mean ± standard deviation (SD).

† Data are presented as median (interquartile range [IQR]).

The patients were asked about the number of weekly erections experienced after the treatment. Spontaneous erection 3 times and above weekly was evaluated as satisfactory ( 12 ).

The addition of hMG therapy to the patients was planned after the 6^th^ month of hCG treatment.

### Biochemical analyses, testicular volume, imaging methods, and bone mineral density measurements

Fasting blood samples of all patients were taken from the antecubital vein in the morning after overnight fasting (at least eight hours). Serum hormone levels were measured by electrochemiluminescence immunoassay (ECLIA) (Elecsys and Cobas Modular analytics E 710, Cobas e 601, Mannheim, Germany). Scrotal ultrasound (Acuson Corporation, Siemens, Mountain view, CA, USA) of the patients was performed with a 7.5 Mhz probe. The normal range of testicle size was determined to be 15-25 mL ( 13 ). Testicular volume was calculated as the median volume of the left and right testes. Testicular volumes were recorded before treatment and at the first year of the treatment. All patients underwent a three-dimensional contrast-enhanced pituitary magnetic resonance imaging (MRI) scan with 1.5 Tesla GE healthcare MRI device (Optima MR360, Milwaukee, Wisconsin, USA). Bone mineral density (BMD) was measured using dual energy X-ray absorptiometry (DEXA - DPX-LUNAR 74400) scan of the lumbar spine. Lumbar BMD (L1-L4 spine) Z-score values were recorded before treatment and at the end of the 6^th^ month. For semen analysis, modern computerized technology (CASA = Computer-Assisted Semen Analysis) in which dynamic parameters are evaluated was used.

### Statistical analysis

Normality of distribution was examined using the Shapiro-Wilk W test. Descriptive statistical methods including percentage and mean ± standard deviation (SD) or median (interquartile range [IQR]) were used to provide the basic characteristics of the data. Wilcoxon signed ranks test was used for non-normally distributed continuous variables for hCG and T treatment groups. Paired samples t-test was used for normally distributed continuous variables. Comparison of the testosterone levels on month 0, 1, 3, and 6 of treatment was carried out using the Friedman test. For the assessment of weekly morning erection frequency, the Yates (Continuity Correction) *χ* 2 test was used. Comparison of data between the two groups was performed using the Mann-Whitney U test (for left-right testicular volume and estradiol) and the independent sample t-test (for total testosterone and T/E ratio). Receiver Operating Characteristic (ROC) curve analysis was performed to estimate the effective T/E ratio in patients with sufficient frequency of spontaneous morning erection. As the result of ROC-curve analysis, the cut-off value of the effective T/E ratio for spontaneous morning erection was determined with the corresponding sensitivity and specificity. Area under the ROC curve (AUC) value with 95% CI was calculated. In post hoc analysis, when effect size (df) = 0.95 and alpha = 0.05, a minimum sample size of 29 and a maximum of 49 was determined to provide a power of 80-95%. Based on these data, G power (14) was found to be 87.5 % for 36 patients in our study. All statistical analyses were carried out using SPSS 23.0 version (IBM Corporation, Armonk, NY, US). When two-tailed p < 0.05, the differences were considered statistically significant.

## RESULTS

### Clinical characteristics

The mean age of the 36 patients included in the study was 28.5 ± 8.7 years and the mean body mass index (BMI) was 25.5 ± 2.7 kg/m². Other demographic, anthropometric, and biochemical characteristics of the patients are listed in Tables 1 and 2 . Fixed dose (250 mg testosterone) was applied in the T treatment group and dose titration was performed in the hCG group ( Table 3 ). Spontaneous morning erection was achieved in patients with sufficient testosterone levels. The initial spermiogram analyses of all patients were azoospermic.

**Table 2:** Demographic, anthropometric, and biochemical characteristics of patients according to treatment groups

Age (Years) *	26.88 ± 6.75	29.95 ± 10.03	0.296 a
BMI (kg/m^2^) *	25.53 ± 2.62	25.58 ±2.83	0.953 a
Hematocrit (%) *	45.40 ± 3.14	42.91 ± 3.35	0.029 a
Total cholesterol (mg/dL)	167.9 ± 27.6	152.1 ± 26.8	0.103 a
HDL-cholesterol (mg/dL)	49.8 ± 10.3	42.8 ± 13.4	0.254 a
LDL-cholesterol (mg/dL)	93.3 ± 21.3	88.7 ± 22.3	0.328 a
Triglyceride (mg/dL)	121.6 ± 67.8	100.2 ± 54.3	0.356 a
FSH (mIU/mL) †	1.3 [2.0]	2.4 [8.8]	0.030 b
LH (mIU/mL) †	0.6 [1.6]	1.8 [6.8]	0.129 b
ACTH (pg/mL) *	28.90 ± 17.81	23.22 ± 10.49	0.246 a
Cortisol (μg/dL) †	13.8 [7.7]	10.6 [9.0]	0.076 b
TSH (μIU/mL) †	1.8 [1.1]	1.6 [1.4]	0.186 b
Prolactin (ng/mL) *	9.10 ± 4.18	10.34 ± 6.51	0.508 a
Mean left testicular volume (mL) †	1.1 [6.3]	1.6 [1.9]	0.510 b
Mean right testicular volume (mL) †	1.0 [5.8]	1.6 [2.5]	0.415 b
Total testosterone (ng/mL) †	36 [31]	60 [86]	0.112 b
Estradiol (pg/mL) *	20.6 ± 2.5	29.9 ± 2.2	0.251 a

hCG: human chorionic gonadotropin; T level: total testosterone level; BMI: body mass index; FSH: follicle-stimulating hormone; LH: luteinizing hormone; ACTH: adrenocorticotropic hormone; TSH: thyroid-stimulating hormone.

a Independent sample t-test.

b Mann-Whitney test.

* Data are presented as mean ± Standard deviation (SD).

† Data are presented as median (interquartile range [IQR]).

**Table 3:** Average dose of hCG therapy during titration

	Before Treatment	1^st^ Month	3^rd^ Month	6^th^ Month
T level in those receiving hCG therapy (ng/dL)	36	220	319	384
Average dose of hCG therapy (IU/week)	-	4,558	6,500	7,264

T level: total testosterone level; hCG: human chorionic gonadotropin.

### Testicular size and bone mineral density

In comparative testicular ultrasonography of patients receiving testosterone therapy, the median right testicular volume was 1.6 [2.5] mm³ before treatment and 1.8 [2.2] mm³ after treatment (p = 0.110) and the increase in left testicular volume was statistically significant (p = 0.008) with 1.6 [1.9] mm³ before treatment and 1.8 [3.4] mm³ after treatment.

Regarding patients on hCG treatment, the increase in volume for both testicles was statistically significant (p < 0.001). The median right testicular volume was 1.0 [5.8] mm³ before treatment and 2.5 [6.5] mm³ (p < 0.001) after treatment. The median left testicular volume was 1.1 [6.3] mm³ before treatment and was 2.8 [7.5] mm³ after treatment.

In L1-L4 BMD, the median Z-score of patients receiving T treatment was -2.9 [1.2] before treatment and -2.2 [0.9] after treatment (p < 0.001). Similarly, for hCG treatment, median Z-score was -2.7 [1.5] before treatment and -2.1 [0.7] after treatment (p < 0.001).

### Testosterone to estradiol ratio

Baseline, 1, 3, and 6 month median testosterone values were 60 [56], 296 [371], 346 [191], and 400 [234] ng/dL, respectively, in patients on T treatment (p < 0.0001). On the other hand, the corresponding median values of testosterone levels in patients receiving hCG treatment were 36 [31], 220 [126], 319 [138] and 384 [220] ng/dL, respectively (p < 0.0001). Serum estradiol, T/E ratio, and number of morning erections are given in Table 4 .

**Table 4:** Pre- and post-treatment differences in patients

	Before testosterone treatment (n: 19)	After testosterone treatment (n: 19)	p	Before hCG treatment (n: 17)	After hCG treatment (n: 17)	p
Total testosterone (ng/dL) †	60 [86]	400 [234]	<0.001 a	36 [31]	384 [220]	<0.001 a
Estradiol (pg/mL) *	29.9 ± 2.2	29.5 ± 2.4	0.149 b	20.6 ± 2.5	40.8 ± 1.9	<0.001 b
Total testosterone to estradiol ratio ([ng/dL]/[pg/mL]) †	1.8 [2.9]	13.3 [9]	<0.00 a	1.6 [1.5]	9.1 [6]	<0.00 a
Morning erections (≥3/week), (n, %)	0 (0%)	10 (58.8%)	0.002 a	0 (0%)	6 (35.3%)	0.014 a
Right Mean testicular volume (mL) †	1.6 [2.5]	1.8 [2.2]	0.110 a	1.0 [5.8]	2.5 [6.5]	<0.001 a
Left Mean testicular volume (mL) †	1.6 [1.9]	1.8 [3.4]	0.008 a	1.1 [6.3]	2.8 [7.5]	<0.001 a
Lumbar Z score †	-2.9 [1.2]	-2.2 [0.9]	<0.001 a	-2.7 [1.5]	-2.1 [0.7]	<0.001 a

hCG: human chorionic gonadotropin.

a Wilcoxon signed ranks test.

b Paired samples t-test.

* Data are presented as mean ± standard deviation (SD).

† Data are presented as median (interquartile range [IQR]).

**Figure 2:**
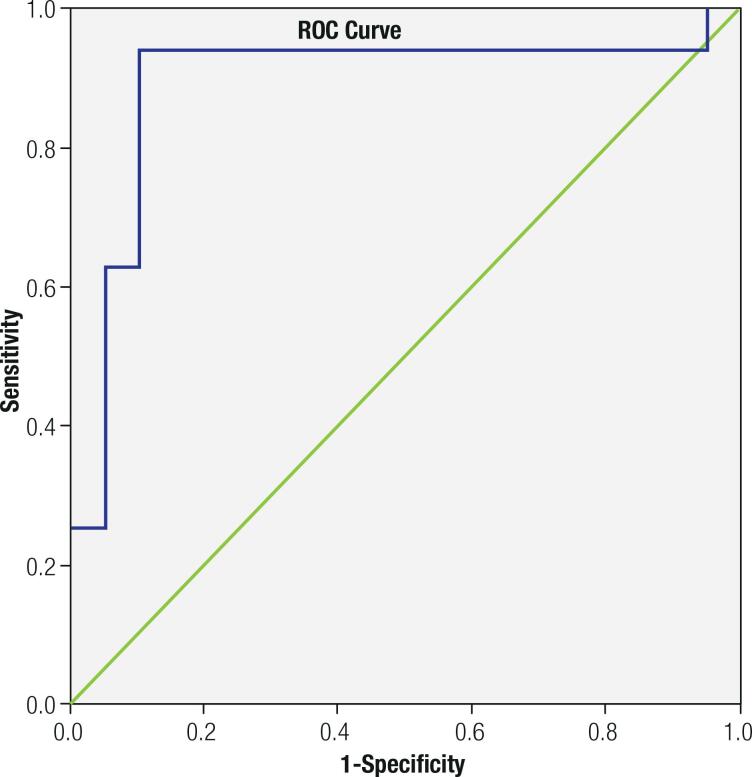
ROC curve analysis for testosterone to estradiol ratio according to spontaneous morning erection frequency.

ROC-curve analysis to estimate the effective testosterone estradiol ratio as a determinant of sufficient weekly spontaneous erection (≥3/week) revealed the cut-off value for T/E ratio as 12.0 (sensitivity 93.8%, specificity 90.0%) ( Figure 2 ). The AUC value for T/E ratio was 0.891 (95% CI: 0.76-1.00).

### Comparison of treatment groups

There was no significant difference between the treatment groups in terms of total testosterone, T/E ratio, or frequency of morning erections (≥3/week) (p > 0.05), although there was a statistically significant difference in terms of median left-right testicular volume and estradiol levels (p < 0.05) ( Table 5 ).

**Table 5:** Comparison of the data of patients receiving testosterone and human chorionic gonadotropin therapy

	Testosterone treatment (n: 19)	hCG treatment (n: 17)	P
Δ Total testosterone (ng/dL) †	345.7 ± 136.0	400.5 ± 151.7	0.264 b
Δ Estradiol (pg/mL) *	-1 [2]	20 [2]	<0.001 a
Morning erections (≥3/week), (n, %) *	10 [52.6%]	6 [35.3%]	0.478 c
Δ Total testosterone to estradiol ratio †	9.4 ± 5.8	8.9 ± 4.4	0.063 b
Δ Left Mean testicular volume (mL) *	0 [0.3]	1.2 [1.5]	<0.001 a
Δ Right Mean testicular volume (mL) *	0 [0.4]	1.3 [1.5]	<0.001 a

hCG: human chorionic gonadotropin.

a Mann-Whitney U test.

b Independent t-test.

c Yates (Continuity Correction) *χ* 2 test.

* Data are presented as median (interquartile range [IQR]).

† Data are presented as mean ± Standard deviation (SD).

hCG: human chorionic gonadotropin; T level: total testosterone level.

### Safety and tolerability

During the follow-up, hCG and T treatments were not found to be associated with serious side effects such as hepatotoxicity, polycythemia, and thromboembolism. Pain and acne occurred in 9 and 4 patients, respectively, in the gluteal area of the injection site after treatment.

## DISCUSSION

This study was performed to determine the effective serum T/E ratio in patients with hypogonadotropic hypogonadism who received hormone replacement therapy and experienced satisfactory spontaneous morning erection (≥3/week). The cut-off value of T/E ratio according to the frequency of spontaneous morning erection was found to be 12.0. To the best of our knowledge, this is the first study to estimate the relationship between T/E ratio and frequency of morning erections in patients with hypogonadotropic hypogonadism.

Male sexual dysfunction, including decreased libido, erectile dysfunction, and abnormal ejaculation is a common type of health disorder ( 15 - 17 ). Three to five spontaneous erections occur in healthy men ( 12 ). Night penile erection (NPE) is a natural phenomenon that is not sexually aroused. The duration of NPE decreases with age, but the frequency does not change ( 18 ). In general, men believe that morning erection is indicative of sexual health ( 19 ). Therefore, the weakness of morning erection can have a negative impact on a man’s sexual life. In this study, it was observed that the weekly frequency of spontaneous morning erection was lower in patients with normal testosterone levels in the hCG treatment than those in the T treatment group. Although the difference was not statistically significant, there was a numerical dominance in favor of T treatment (58.8 % *vs.* 35%).

Estradiol, which until recently had been defined only as a female hormone, has been reported to play a role in male sexual functions ( 20 ). As testosterone levels decrease and estradiol levels rise, the ratio of free testosterone to estradiol reaches a critical point and the gonadotropin-suppressing effects of estrogen become dominant ( 21 ). Since androgens are the precursors of estrogens and the main source of estrogen production in men occurs through peripheral aromatization, replacing testosterone can also mean replenishing estradiol. Since the patients included in our study were non-obese (BMI < 30 kg/m^2^), peripheral aromatization as the source of estrogen probably took little part. This probably explains the stable levels of estradiol observed in the T group after treatment. Concerning the hCG treatment, it can be stated that the elevation of estradiol was an expected result due to the effect of treatment on testicular steroidogenesis ( 22 ).

El Sakka advocated the relationship between low testosterone and/or high estradiol levels and erectile dysfunction ( 23 ). According to his research, low testosterone levels were considered the main cause of erectile dysfunction. However, the simultaneous presence of high estradiol was thought to increase the severity of erectile dysfunction. In line with this, the data obtained in our study showed that, even though total testosterone levels were increased to normal range under treatment, less patients achieved spontaneous morning erection in the hCG treatment group than those in the T group, possibly due to the high estradiol levels in the former.

According to a recently published study, it was shown that both low testosterone levels and high estradiol levels had negative effects on erectile function and these correlated with the severity of erectile dysfunction. It has been speculated that the T/E ratio may be a useful indicator for the follow-up of patients with erectile dysfunction ( 24 ). Even high estradiol levels are accepted as an independent risk factor for erectile dysfunction ( 25 , 26 ). Besides, one study on sexual dysfunction revealed that changes in estradiol and testosterone levels had effects on premature ejaculation other than erectile dysfunction ( 27 ).

Here, the frequency of spontaneous morning erections increased significantly when the cut-off value of the T/E ratio after treatment was specified as 12.0 (sensitivity 93.8%, specificity 90.0%). To the best of our knowledge, there is no study in the literature that directly examines the association between T/E ratio and frequency of erection in hypogonadotropic hypogonadism. Pavlovich and cols. explored the relation between T/E ratio and fertility and found that infertile individuals had a lower T/E ratio than fertile individuals, at 14.5 in fertile cases versus 6.9 in infertile cases ( 28 ). The authors also reported that T/E ratio increased from 5.0 to 12.7 after treatment in patients who received an aromatase inhibitor. Although we found an association between T/E ratio and spontaneous morning erection, the findings of Pavlovich and cols. may provide insight into future fertilization as a predictive value.

In the present study, testicular volumes of patients were significantly increased during the course of treatment. Among these, the increase was statistically significant in the hCG group but not in patients undergoing testosterone replacement. This significant increase in the hCG group was compatible with the previous literature. This result shows once again the positive effect of hCG treatment on testicular volume ( 29 ).

Due to the retrospective and single-left design, bias of selection is our main limitation. The second limitation is the relatively small sample size and the lack of a control group.

In conclusion, in patients with hypogonadism who are under treatment, elevated estradiol-induced erectile dysfunction symptoms may persist even if serum testosterone levels are normal. Keeping testosterone to estradiol ratio around 12.0 may increase the frequency of spontaneous erections in the morning. To better understand the role of the T/E ratio in this disease, randomized double blind placebo control (RDBPC) studies involving large numbers of patients are needed.
